# Crystal structure of the DdrB/ssDNA complex from *Deinococcus radiodurans* reveals a DNA binding surface involving higher-order oligomeric states

**DOI:** 10.1093/nar/gkt759

**Published:** 2013-08-23

**Authors:** Seiji N. Sugiman-Marangos, John K. Peel, Yoni M. Weiss, Rodolfo Ghirlando, Murray S. Junop

**Affiliations:** ^1^Department of Biochemistry and Biomedical Sciences and M. G. DeGroote Institute for Infectious Disease Research, McMaster University, 1200 Main Street West, Hamilton, Ontario L8N 3Z5, Canada and ^2^Laboratory of Molecular Biology, National Institute of Diabetes and Digestive and Kidney Diseases, National Institutes of Health, 5 Center Drive, Bethesda, MD 20892, USA

## Abstract

The ability of *Deinococcus radiodurans* to recover from extensive DNA damage is due in part to its ability to efficiently repair its genome, even following severe fragmentation by hundreds of double-strand breaks. The single-strand annealing pathway plays an important role early during the recovery process, making use of a protein, DdrB, shown to greatly stimulate ssDNA annealing. Here, we report the structure of DdrB bound to ssDNA to 2.3 Å. Pentameric DdrB was found to assemble into higher-order structures that coat ssDNA. To gain further mechanistic insight into the protein's function, a number of point mutants were generated altering both DNA binding and higher order oligomerization. This work not only identifies higher-order DdrB associations but also suggests the presence of an extended DNA binding surface running along the ‘top’ surface of a DdrB pentamer and continuing down between two individual subunits of the ring structure. Together this work sheds new insight into possible mechanisms for DdrB function in which higher-order assemblies of DdrB pentamers assist in the pairing of complementary ssDNA using an extended DNA binding surface.

## INTRODUCTION

*Deinococcus radiodurans* is renowned for its ability to recover from exposure to extreme ionizing radiation (IR), desiccation, ultraviolet radiation and a variety of DNA damage-inducing agents. Its capacity to withstand these various forms of damage has been attributed to a combination of protection of the proteome by free-radical scavenging manganese complexes (1–3) and an efficient DNA repair system involving several proteins found uniquely in *Deinococcus* (2, 4–6).

This contingent of novel proteins includes DdrB, which was identified as highly up-regulated following DNA damage by two independent experiments monitoring mRNA transcript levels in *D. radiodurans* recovering from acute IR exposure (4,7). This has recently been corroborated by mass spectrometry-based proteomic analysis of *D. radiodurans* post-IR, which showed that DdrB is the second most abundant DNA repair protein during recovery (8). Further underlining its involvement in DNA damage recovery, *D. radiodurans* Δ*ddrB* is radiosensitive, experiencing a decrease in survival following high doses of IR and delayed recovery at low doses (4,9).

Owing to its abundance in the cell post-IR (8), its ability to bind ssDNA *in vitro* (10,11) and a disordered C-terminus, DdrB has been implicated as a stress-inducible functional equivalent to canonical single-stranded binding protein (SSB) (10). It has since been demonstrated, however, that the purported SSB-like C-terminal motif is not necessary for radioresistance (9) and that DdrB possesses novel activities not shared by SSB, such as the ability to promote annealing of complementary oligonucleotides *in vitro* (12)*,* and suppress, rather than stimulate, RecJ exonuclease activity (13). The crystal structure of DdrB confirmed that in addition to be being functionally dissimilar to canonical SSB, DdrB is structurally distinct and likely does not bind ssDNA in the same way as SSB, as it lacks an OB-fold (11).

Under extreme damaging conditions, hundreds of double-strand breaks (DSB) are generated and are repaired in *D. radiodurans* by two main pathways, single-strand annealing (SSA) and extended synthesis-dependent strand annealing (ESDSA) (2,6). Following fragmentation of the genomic DNA, the resulting segments of dsDNA are processed by 5′ exonucleases, yielding 3′ overhangs. These overhangs are then either annealed directly to complementary strands yielding larger dsDNA fragments by SSA, or undergo RecA-mediated strand-invasion of homologous duplexes by ESDSA (6). SSA appears to function independently of RecA and is thought to play a role early during DSB repair in *Deinococcus* spp., particularly when the number of strand breakages is extensive. It has been suggested that DdrB may play an important role in SSA due to its ssDNA annealing activity (9), similar to Rad52 in the analogous eukaryotic pathway.

Visualization by electron microscopy revealed that DdrB coats ssDNA like ‘beads on a string’ similar to both SSB and RecA (10), and intrinsic fluorescence quenching titration demonstrated the binding stoichiometry of the complex as between 41 and 56 nucleotides per pentamer (9). Here, we have further characterized the interaction between DdrB and ssDNA. We report the X-ray structure of DdrB in complex with ssDNA to 2.3 Å and assessed the DNA binding activity of a number of point mutants generated based on this structure by fluorescence polarization. This analysis not only confirmed the ssDNA–protein interaction observed within the crystal structure, but further suggested the presence of an extension of this surface continuing along the ‘top’ face of the DdrB pentamer. Additionally, DdrB was found to mediate ssDNA coating through assembly of a higher-order structure involving two DdrB pentamers. These protein–protein interactions were verified in solution using analytical ultracentrifugation (AUC). Based on these results, we propose potential mechanisms for how DdrB promotes observed ssDNA annealing.

## MATERIALS AND METHODS

### Protein expression and purification

DdrB from *D. radiodurans* (DdrB_Dr_) was synthesized and sub-cloned into pPROEX-HT by Geneart, producing the expression construct MJ4730. MJ4748 was then generated by amplifying DdrB_Dr_ from MJ4730, introducing a stop codon following residue 144. This gene product was then cloned into the expression vector pET151/D-Topo (Invitrogen) per the manufacturer's protocol (11). DdrB_1__–144_ was expressed and purified as described previously with the following amendments: (i) protein used in crystallography had the 6His tag removed by cleavage with TEV protease and was exchanged into 20 mM Tris pH 6.0, 100 mM KCl; (ii) protein used in DNA binding and AUC experiments was not treated with TEV to remove the N-terminal 6His tag. DdrB mutants were generated using the Maxime PCR PreMix (i-pfu) kit from iNtRON Biotechnology as per the manufacturer’s protocol. All mutants were verified by sequence analysis.

### Structure determination

Crystals were grown by the hanging-drop vapour diffusion method at 20°C. A 1.5 µl DdrB_Dr_/ssDNA solution (740 µM DdrB_Dr_ and 160 µM 50b poly dT (Integrated DNA Technologies) in 20 mM Tris pH 6.0, 100 mM KCl) was mixed with 1.5 µl of crystallization buffer (50 mM MES pH 5.6, 300 mM KCl, 10 mM MgCl_2_, 5% PEG 8000) and dehydrated over 250 µl of 1.35 M (NH_4_)_2_SO_4_. A description of difficulties encountered during crystallization has been published elsewhere (14). Diffraction data were collected at the NSLS X29A beamline at Brookhaven National Laboratory (NY, USA). The dataset was processed and scaled to 2.30 Å with HKL2000 (15), and solved by molecular replacement with Phenix-AutoMR (16) using the apo-structure of DdrB_Dg_ (PDBID 4EXW) as a search model. ssDNA was built into the structure manually with Coot (17), and structure refinement was carried out through multiple iterations of manual refinement in Coot and automated refinement with Phenix-AutoMR until R and R_free_ values converged and geometry statistics reached an appropriate range (Table 1). Model coordinates and experimental data have been submitted to the Protein Databank (PDB) under the accession code: 4HQB.

**Table 1.** Data collection and model refinement statistics

Data collection		Model and refinement	
Space group	P3_2_	Resolution (Å)	40.30–2.30
Unit cell parameters		*R*_work_/*R*_free_ (%)	19.0/24.6
a,b,c (Å)	110.7, 110.7, 58.8	Reflections_observed_	35 711
Matthews coefficient	2.61	Reflections_Rfree_	1798
Molecules in ASU	6	No. atoms	
Resolution range (Å)^a^	50.0–2.30 (2.34–2.30)	Protein	4703
Observed reflections	203 021	DNA	160
Unique reflections^a^	35 736 (1844)	Water	194
Redundancy^a^	5.7 (5.6)	R.m.s.d. bond	
Completeness (%)^a^	99.9 (100.0)	Lengths (Å)	0.008
I/σ(I)^a^	17.4 (2.3)	Angles (°)	1.07
*R*_merge_ (%)^a^	6.8 (72.6)	Average B-factor (Å^2^)	61.3
Wilson B-factor (Å^2^)	52.34	Protein	61.6
		DNA	63.4
PDB accession code	4HQB	Water	52.0

^a^Statistics for the highest resolution shell are shown in parentheses.

### Structure analysis

Analysis of protein–protein interfaces was performed using the PISA server from PDBe (18). Assessment of protein–ssDNA interactions was carried out with the aid of NUCPLOT (19) and BINANA 1.0.0 (20). Input files for BINANA in pdbqt format were generated with AutoDockTools (21) using calculated Gasteiger charges and merged non-polar hydrogens.

### DNA binding

Gel-shift assays were performed in 20 µl of EMSA buffer (20 mM Tris pH 8.0, 100 mM KCl, 15% (v/v) glycerol) with 10 µM of a 50b poly dT substrate and increasing concentrations of DdrB pentamer (0, 2, 10, 20, 50, 100 µM). Samples were resolved by electrophoresis on 4–20% polyacrylamide TGX precast gels (Bio-Rad) at 100 V for 90 min and visualized by SYBR Gold (Invitrogen) staining. For fluorescence polarization experiments, a 20 b poly dT substrate with a 5′ 6-FAM label (200 nM) was titrated with increasing concentrations of DdrB pentamer (0, 0.1, 0.2, 1, 2, 5, 10, 15, 20, 40 µM) in a total volume of 50 µl of binding buffer (20 mM Tris pH 8.0, 100 mM KCl). Fluorescence polarization measurements were performed in black, clear-bottom 96-well plates using a BioTek Synergy 4 Hybrid Microplate Reader (sensitivity = 0.35) using excitation and emission wavelengths of 485 and 528 nm, respectively. Fluorescence anisotropy (A) was calculated from polarization measurements (A = 2 P/(3 − P)). Fluorescence anisotropy binding data were modeled in terms of an A + B = AB isotherm, where A represents the 20 b dT and B the DdrB pentamer, in SEDPHAT 10.51 (22). Errors reported for the dissociation constant K_d_ represent 95% confidence intervals. All DNA substrates were purchased from Integrated DNA Technologies.

### Sedimentation velocity analytical ultracentrifugation

Stock solutions of the wild-type DdrB, E51A and R83A mutants were obtained in 100 mM KCl and 20 mM Tris pH 8.0. These were used to prepare samples for sedimentation velocity carried out at different loading concentrations, ranging from 10 µM to 0.64 mM. High concentration samples (0.04–0.64 mM) were loaded into 3-mm 2-channel epon centerpiece cells (100 µl), whereas low concentration samples (10–20 µM) were loaded into 12-mm 2-channel epon centerpiece cells (400 µl). Sedimentation velocity experiments were conducted at 20°C and 42 krpm on a Beckman Coulter ProteomeLab XL-I analytical ultracentrifuge using both the absorbance (280 nm) and Rayleigh interference optical systems. Time-corrected data (23) were analysed in SEDFIT 14.3e (24) in terms of a continuous *c(s)* distribution covering an *s* range of 0.0–35.0 S with a resolution of 350 and a confidence level (F-ratio) of 0.68 with a maximum entropy regularization. Excellent fits were obtained with r.m.s.d. values ranging from 0.002–0.012 fringes or 0.003–0.007 absorbance units. The solution density (ρ) and viscosity (η) were calculated based on the solvent composition using SEDNTERP 1.09 (25,26). The protein partial specific volumes *v* were calculated based on the amino acid composition using SEDNTERP 1.09 (25,26), and sedimentation coefficients were corrected to standard conditions *s_20__,w_*. To estimate the proportions of each of the contributing species, sedimentation velocity data were further analysed in SEDPHAT 10.51 (27) in terms of a set of non-interacting discrete species corresponding to 1, 2, 3, 4 and 5-mers of the DdrB pentamer.

## RESULTS AND DISCUSSION

### The co-crystal structure of DdrB bound to ssDNA

**Figure 1.**
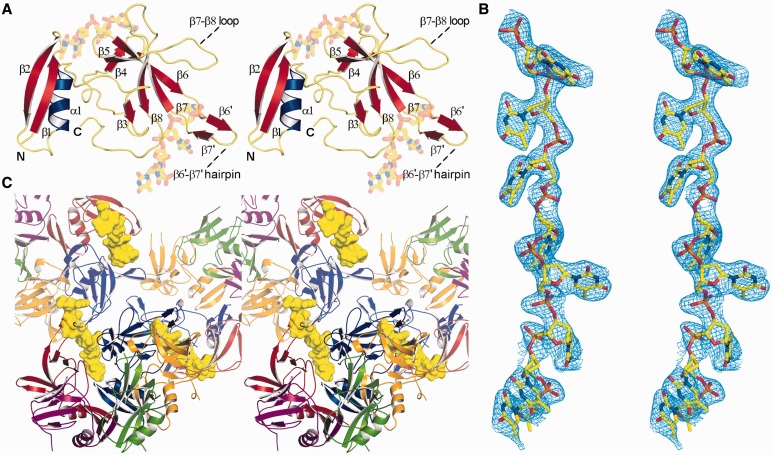
Structure of the DdrB_1–144_ from *D. radiodurans* bound to ssDNA (stereo-images). (**A**) DdrB monomer bound to two 4-mers of ssDNA. (**B**) ‘Kicked’ 2Fo-Fc OMIT map (calculated with DNA removed), illustrating the electron density of the bound ssDNA. (**C**) Three DdrB pentamers. The 8b DNA molecule (yellow) bridges symmetrically related pentamers (displayed in partial transparency). This figure (and figures 2, 3, 6 and 7) was prepared using PyMol (http://www.pymol.org/).

The structure of DdrB_Dr_ bound to ssDNA (Figure 1A) was solved by molecular replacement using the apo-structure of DdrB_Dg_ (PDBID: 4EXW) as a search model. Molecular replacement produced a good quality electron density map for the entire asymmetric unit (one DdrB_Dr_ pentamer) with very clear density for bound ssDNA in two clefts formed between three DdrB subunits. In total, eight bases of dT could be modelled into the density (Figure 1B), which appear to form a continuous chain through the crystal when crystallographic symmetry is applied (Figure 1C). Although there are five channels formed between adjacent monomers within a single pentamer, only two are occupied by DNA. This may simply reflect constraints imposed by crystal packing. Alternatively, such an arrangement may be required for function in ssDNA annealing. Consistent with this possibility, the chemical environment surrounding each DNA base, observed in both occupied channels, is unique (described in detail below).

The overall structures of apo DdrB_Dg_ and ssDNA bound DdrB_Dr_ do not deviate to a great degree, with two notable exceptions. The regions joining β6–β7 and β7–β8 (L_β7__–β8_), which were unstructured in the apo model, are stabilized through interactions with ssDNA and are now visible in the electron density (Supplementary Figure S1). Due to high B-factors, disordered side chains and poor connectivity in the electron density, the original structure of DdrB_Dg_ deposited to the PDB was misnumbered in the latter portion of the protein. The sequence joining β6–β7 contains a β-hairpin comprising two short β-strands spanning residues 89–91 (β6′) and 94–96 (β7′), and a short loop (R92 and K93) (Figure 1A). The β6′–β7′ hairpin is involved in direct interactions with ssDNA in the DNA-bound structure, as well as playing a key role in mediating the oligomeric assembly observed within the crystal lattice. Similar to the apo structure, this segment is not visible in the electron density in the two subunits of DdrB that are void of interactions with ssDNA. Through structural superposition with DdrB_Dr_, the amino acid assignment in the structure of DdrB_Dg_ has been amended and the revised structure has been deposited to the PDB under PDBID: 4EXW, superseding the previous entry. A secondary structure topology diagram is presented in Supplementary Figure S3.

### DNA binding residues

**Figure 2.**
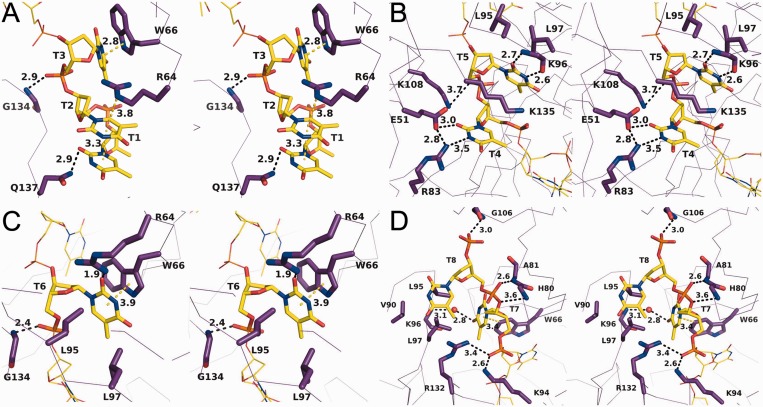
Stereo-images of DdrB interactions with T1–T3 (**A**), T4–T5 (**B**), T6 (**C**), T7–T8 (**D**). Protein is represented in C_α_ form with highlighted residues and ssDNA represented in stick. Amino acid residues (yellow), DNA bases (purple) and interaction distances are labeled (Å). Polar interactions and π-interactions are denoted by black and yellow dashed lines, respectively.

In the structure, eight DNA bases were bound to a single pentamer. The coordinates for this model have been deposited with two individual 4-mers of dT bound between subunits E/A (T1–T4) and A/B (T5–T8); however, when crystallographic symmetry is applied, the two 4-mers form a continuous 8-mer related by the symmetry operation [−Y, X–Y, Z + 2/3]. For purposes of clarity, the binding between DdrB and ssDNA will be described for the unbroken 8-mer of ssDNA as it passes between chains A and E in the ‘first’ pentamer, to the groove formed between chains A and B in the ‘second’ pentamer (referred to as chains A’ and B’ hereafter). The first three bases of dT interact exclusively with residues from chains A and E, and run in the 5′->3′ direction in the cleft between the two subunits from the ‘bottom’ face of the pentamer towards the ‘top’ surface (Figure 2A). This binding channel is flanked on one side by β3–β5 from chain A, and L_β7__–β8_ and the C-terminal coil from chain E on the other. Nucleotides T1 and T2 base-stack and form a planar array through a cation–π interaction with R64_A_ from β3, with T2 sandwiched between T1 and the guanidinium group of R64_A_. The nucleobase of T1 also forms a hydrogen bond with Q137_E_. The nucleobase of T3 forms hydrogen bonds with the guanidinium group of R64_A_, and a π–π interaction with the indole ring of W66_A_ from β3. T3 is further stabilized by a hydrogen bond between the 5′-phosphate and the backbone amine of G134_E_.

Bases T4–T6 bridge the two pentamers, interacting with a surface delineated by: β5, β6 and L_β7__–β8_ from chain A; the β6′–β7′ hairpin and β8 from chain E; α2 and the C-terminal coil from chain A’; and β4 from chain B’ (Figure 2B). T4 is stabilized through electrostatic interactions with R83_A_ and E51_A’_, which also form a salt bridge between them. T5 forms hydrogen bonds between its nucleobase and the backbone amino and carboxylic groups of K96_E_, as well as significant van der Waals (vdW) contacts with the aliphatic portion of K135_A’_. T5 is further stabilized through vdW interactions with the hydrophobic patch on the β6′–β7′ hairpin (L95_E_ and L97_E_), and electrostatic interactions between K108_A_ from L_β7__–__β8_ and the 5′-phosphate. The nucleobase of T6 forms a hydrogen bond with the guanidinium group of R64_B’_, vdW interactions with L95_E_ and L97_E_, and a π–π interaction with the indole ring of W66_B’_. The 5′-phosphate of T6 also forms a hydrogen bond with the backbone amine of G134_A’_ (Figure 2C).

Bases T7 and T8 emerge from the binding channel at the ‘top’ face of the second pentamer (Figure 2D). The 5′-phosphate of T7 forms hydrogen bonds with the ε-amino group of K94_E_ and the guanidinium group of R132_A’_. The nucleobase of T7 forms a t-shaped π–π interaction with the indole ring of W66_B’_ and is further stabilized by a solvent-mediated hydrogen bond network with the backbone of K96_A’_, and vdW interactions with L95_A’_ and L97_A’_. This hydrophobic patch on the β6′–β7′ hairpin, completed by V90_A’_, is also a key factor in stabilizing the nucleobase of T8. The phosphate groups of T8 are stabilized entirely by hydrogen bonding with the backbone amines of A81_B’_, H80_B’_ and G106_B’_. Residues involved in DNA interaction are among the most highly conserved within DdrB. A multiple sequence alignment of DdrB homologues highlighting residues involved in DNA interaction is provided in Supplementary Figure S2.

In accordance with DdrB DNA binding studies published to date, which have used a variety of substrates interchangeably (9–12), the interactions observed between DdrB and DNA within the crystal structure suggest a non-specific mode of binding. Overall, the interactions between DdrB and the bound ssDNA consist largely of charged interactions with the phosphate backbone, and interactions with the planar surfaces of the nucleobases. W66 in particular is involved in stabilizing three nucleobases (T3, T6 and T7) through both face–face and T-shaped π–π interactions. The limited number of hydrogen bonding interactions with the nucleobases, further points to a lack in sequence specificity within the binding channel. Consistent with this interpretation, DdrB crystals of similar morphology could also be obtained using identical crystallization conditions with poly dA ssDNA.

### Quaternary structure

**Figure 3.**
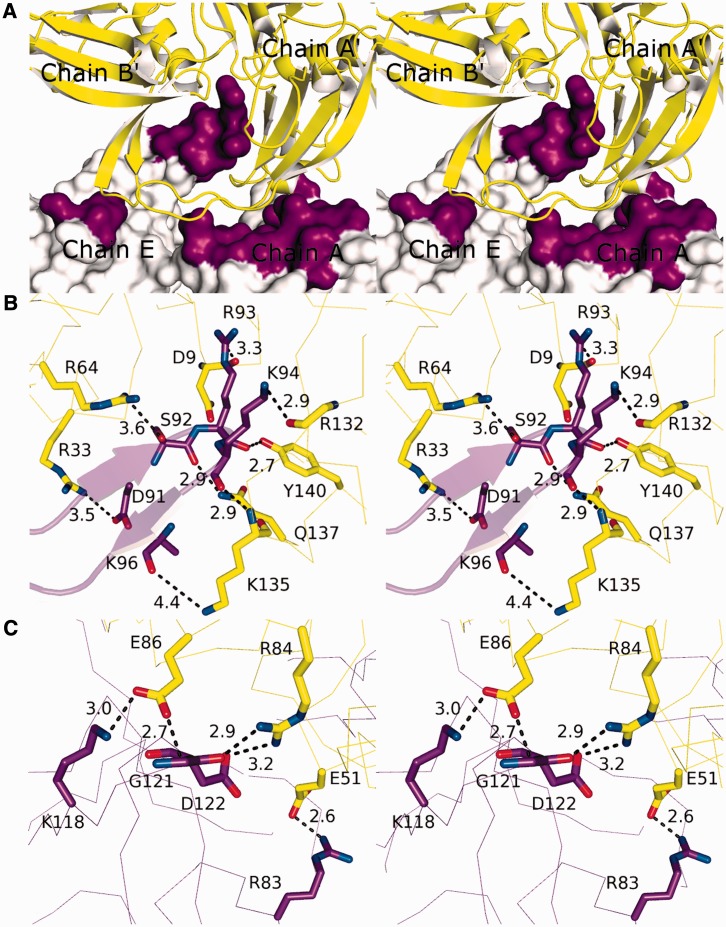
DdrB quaternary interactions (stereo-images). (**A**) The pentamer–pentamer interface. Pentamer ‘1’ (purple) interfaces with pentamer ‘2’ (yellow) through contact surfaces distributed across four chains. (**B**) Close-up of the major interactions formed between the β6′–β7′ hairpin of chain E (purple), inserted into the cleft formed between chains A’ and B’ (yellow). (**C**) Closeup of the major interactions formed between chains A (purple) and A’ (yellow). Distances denoted with dashes represent polar interactions (electrostatics + H-bonds).

Interaction with the continuous 8-mer is coordinated by two pentamers that form an extensive protein–protein interface (Figure 3A) stabilized by numerous electrostatic and hydrogen bonding interactions, and significant buried surface area (1082 Å^2^ as calculated by the PISA server). Dimerization of DdrB pentamers is therefore required to generate the DNA binding channel occupied within the crystal structure. This interface is made up of two major contacts, insertion of the β6′–β7′ hairpin from chain E into the cleft formed between chains A’ and B’ (Figure 3B), and three salt-bridges formed between chains A and A’ (Figure 3C). Among these numerous interactions, the salt-bridge formed between residues E51 and R83 appeared to be a particularly strong interaction.

**Figure 4.**
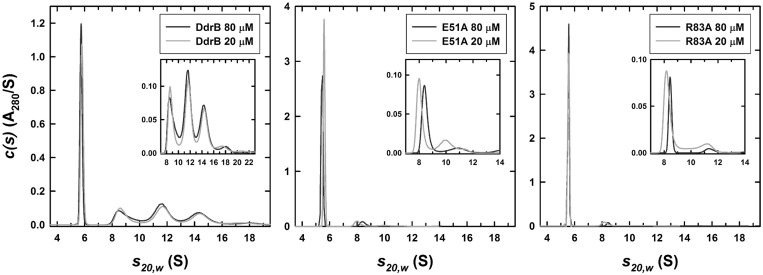
Sedimentation velocity analytical ultracentrifugation. Absorbance *c(s)* profiles for wild-type, E51A and R83A DdrB at loading concentrations of 20 and 80 µM. Similar profiles were obtained using the interference optical detection system. In addition similar profiles were observed for wild-type DdrB at 0.32 and 0.64 mM. A sedimentation coefficient of 5.76 S was obtained for the wild-type DdrB pentamer in a linear extrapolation of the sedimentation coefficient to zero concentration. The best-fit molar mass for this species was 118 ± 6 kDa, consistent with a DdrB pentamer. Values obtained for the E51A pentamer were 5.64 S and 116 ± 6 kDa, values for the R83A pentamer were 5.57 S and 111 ± 5 kDa. Insets expand the *c(s)* profiles to highlight contributions from the decamer (∼9.1 S), penta-decamer (∼11.8 S), didecamer (∼14.6 S) and higher species.

To verify the assembly of higher-order complexes and evaluate potential significance of this assembly in DNA binding, a mutant (E51A) targeting the pentamer self-association interface was generated. Sedimentation velocity AUC was performed with purified recombinant wild-type DdrB and the E51A mutant at 10, 20, 40, 80 and 160 µM protein concentrations utilizing both the absorbance (280 nm) and interference (655 nm) optical detection systems. *C(s)* profiles for wild-type DdrB were consistent with the assembly of pentamers, decamers, penta-decamers and didecamers (Figure 4). While the major species in all concentrations assayed was the pentamer, contributing 39 ± 4% to the total signal, based on an assessment of the relative signal contributions, higher-order assemblies of DdrB made up the remaining 61% of the distribution in solution. The E51A mutant, however, displayed *c(s)* profiles representative of primarily a pentameric assembly, with this species accounting for 88 ± 2% of the total signal (Figure 4). To corroborate this finding, an additional mutant (R83A) was similarly analysed. Like E51A, R83A greatly reduced higher-order assembly, resulting in 92 ± 3% contribution from the single pentameric species (Figure 4). As both E51A and R83A mutants are deficient in their ability to assemble into larger complexes, the interface observed in the crystal packing appears to reflect a quaternary assembly that is relevant in solution. Importantly, this analysis clearly demonstrates that DNA binding is not required for formation of higher-order DdrB pentamer complexes.

**Figure 5.**
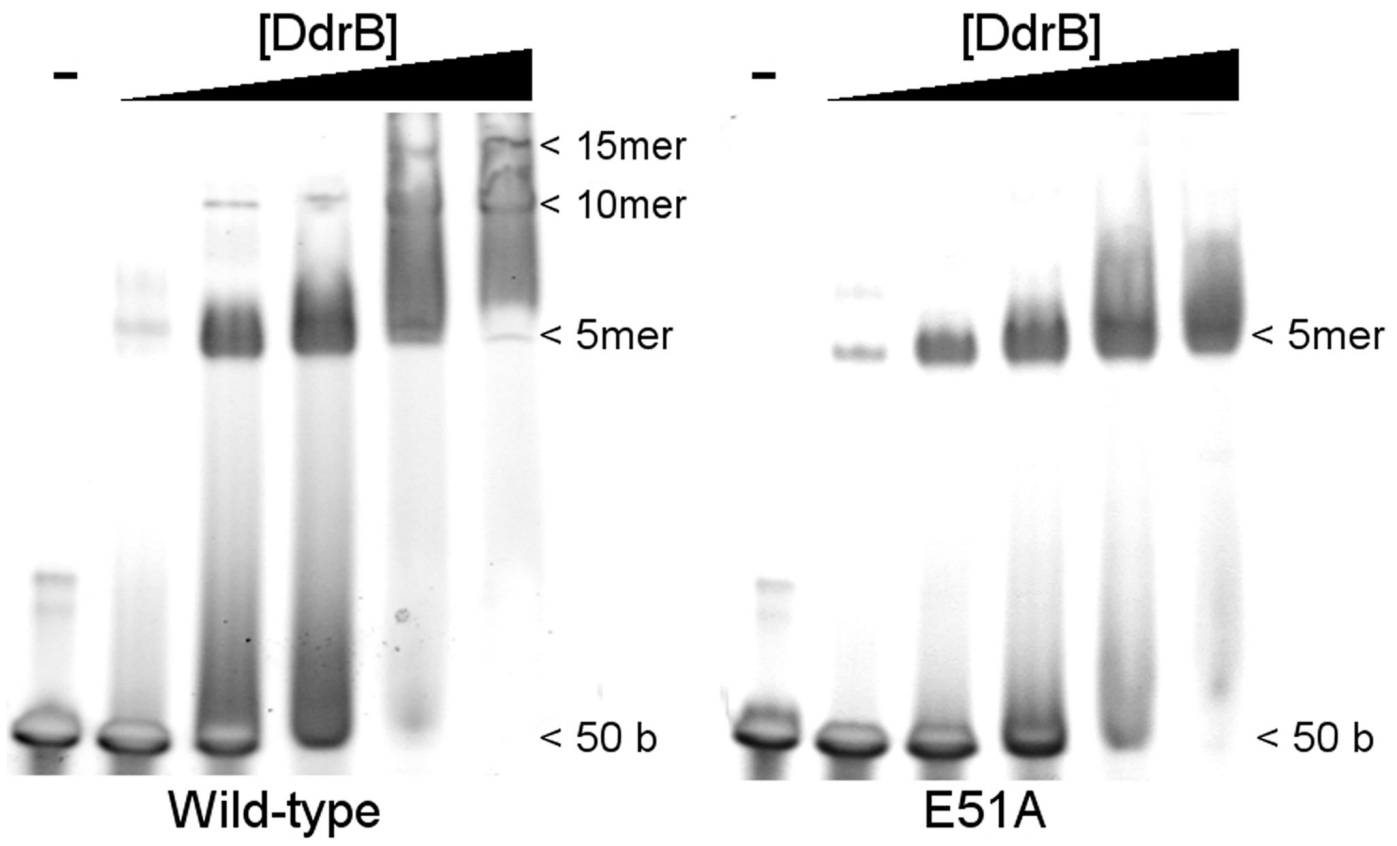
Electrophoretic shift assay of 50b ssDNA by wild-type and E51A DdrB. DNA (10 µM) was incubated with increasing concentrations of DdrB pentamer (0, 2, 10, 20, 50, 100 µM) and resolved on a 4–20% native polyacrylamide gel. Shifts corresponding to DNA-pentamer, DNA-decamer and DNA-pentadecamer nucleoprotein complexes are visible with wild-type DdrB. While the E51A mutant readily forms the DNA-pentamer complex, no distinct shifts corresponding to higher-order assemblies are visible in the gel.

Despite DNA having no obvious effect on the ability of DdrB pentamers to oligomerize, disruption of higher-order assemblies would be expected to disrupt DNA binding, as this surface/channel is formed precisely at the pentamer–pentamer interface. To investigate this possibility, DNA binding of the E51A mutant was assessed using a 50 base DNA substrate. As predicted from the crystal structure, wild-type DdrB was able to form a series of higher-order protein–DNA complexes (e.g. pentamer, decamer, penta-decamer) when resolved on a native polyacrylamide gel (Figure 5). Interestingly, while the initial binding event (single DNA–pentamer complex) was unaffected by disruption of higher-order oligomerization, E51A was unable to generate more than a single nucleoprotein complex (Figure 5). The fact that disrupting oligomerization had no apparent effect on DNA binding affinity suggests that pentamer multimerization may not be required for DNA binding. The presence of a single species with the E51A mutant is still somewhat surprising. If a single DdrB pentamer were still able to bind DNA at the surface observed within the crystal structure, it would only require four bases and therefore with a 50 base oligomer one would have expected multiple individual pentamer binding events and subsequent shifts to have been observed. A more direct interpretation of this result is that although disruption of pentamer oligomerization disrupts the observed DNA binding surface/channel the single pentamer retains DNA binding function. This would imply the existence of an additional unidentified DNA binding surface and would help to reconcile the crystal structure with earlier findings that suggested the size of DNA required to fully saturate DdrB is 40–50 bases (9).

### An extended ssDNA binding surface

**Figure 6.**
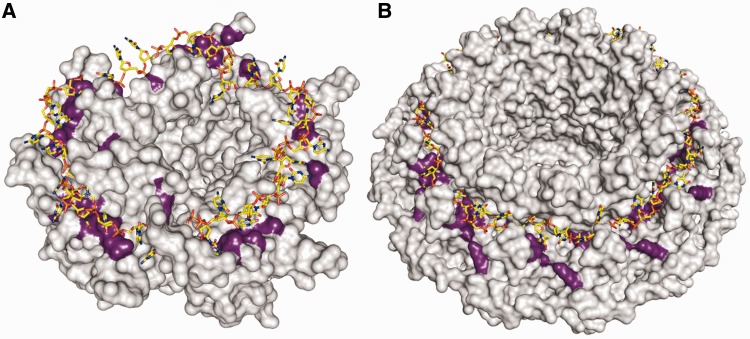
Comparison of models of Rad52 and DdrB ssDNA interactions. (**A**) Model of ssDNA binding utilizing the ‘top’ face of the DdrB pentamer (11). Residues predicted to mediate DNA binding are coloured in purple with ssDNA overlaid for reference. (**B**) The structure of undecameric Rad52 (PDBID—1KN0) with predicted DNA binding interface highlighted in purple. ssDNA is overlaid on the structure of Rad52 (as reported by Singleton *et al.*) (28).

It was proposed previously that DdrB might interact with ssDNA through a surface formed along one continuous (top) face of the pentamer (11) (Figure 6A). This was suggested based on the chemical nature of side chains along this surface, and the structural ‘analogy’ to the ssDNA binding surface of SSB. Interestingly, Rad52 has also been predicted to bind ssDNA along a similar surface formed by a ring assembly (28,29) (Figure 6B). In DdrB, several residues predicted to be involved in ssDNA binding, which lie along the ‘top’ face of the extended β-sheet region (W66, R64, R83), do play key roles in binding ssDNA within the crystal structure. The convex shape of the β-sheets allows the ends to curve inward, forming channels between adjacent monomers. In the crystal structure, the bound ssDNA passes through this channel from one pentamer to another, rather than continuing along the surface of the β-sheet. The possibility therefore exists that DdrB possesses an extended DNA binding surface, continuing from the channel and running along the entire ‘top’ surface (Figure 6A). Evidence for such a scenario was recently reported for uracil-DNA glycosylase that demonstrated an extended DNA binding surface, not observed within the crystal structure (30). Authors suggested that due to constraints imposed on protein–DNA complexes during crystallization, many similar cases might exist in which the DNA binding interface observed within a crystal structure only partially reflects the true biological surface.

**Figure 7.**
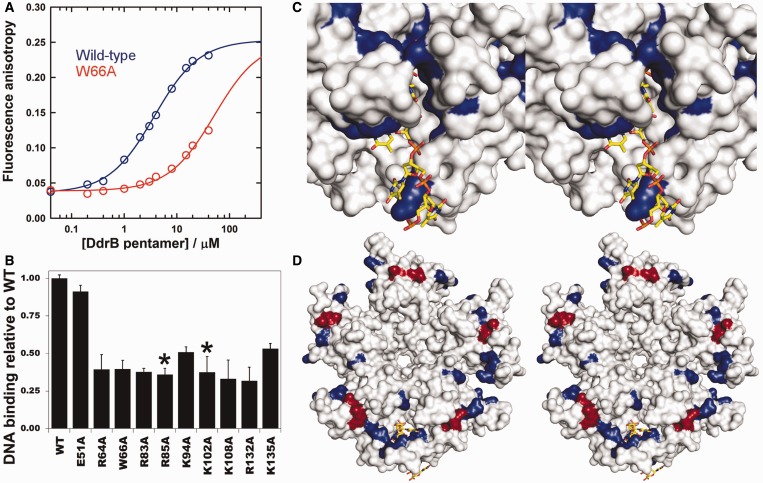
DdrB–ssDNA interactions. (**A**) Titration of 20b FAM labelled dT with increasing concentrations of wild-type (blue) and W66A (red) DdrB analysed by fluorescence anisotropy. The fluorescence anisotropy of the labeled ssDNA substrate increases as its rotational movement decreases on protein binding. Best-fits to a reversible A (DNA) + B (DdrB pentamer) = AB model, shown as solid lines, return K_d_ values of 3.6 ± 0.6 µM for the wild-type DdrB and 51 ± 9 µM for the W66A mutant. In the case of the latter, the saturating anisotropy was fixed at the value obtained for the wild-type DdrB. (**B**) DNA binding of DdrB mutants relative to wild-type DdrB at 3 µM pentamer concentration (error bars represent standard deviation of n = 3 trials). Residues highlighted with a (*) were not observed to interact with ssDNA in the co-crystal structure. (**C**) Stereo-image of the ssDNA binding surface of DdrB from the crystal-structure. Surfaces coloured in blue represent residues that were subjected to amino acid substitution and displayed decreased binding relative to wild-type. (**D**) Stereo-image of the ‘top’ face of the DdrB pentamer. Coloured surfaces represent residues subjected to amino acid substitution, which displayed decreased binding relative to wild-type. Residues coloured in blue were observed to form interaction with ssDNA in the crystal structure, while those in red (R85, K102) showed no interaction with ssDNA. The coloured (blue and red) surface defines a possible extended ssDNA binding mode in addition to the one observed within the structure.

To explore this possibility, alanine substitutions were generated both at residues forming interactions with ssDNA in the crystal structure (R64, W66, R83, K94, K108, R132, K135) and residues that may mediate binding along the ‘top’ surface (R85, K102). A fluorescence polarization-based assay was used to determine an estimate of the K_d_ for the interaction between a 20b poly dT ssDNA substrate and wild-type DdrB pentamer and was found to be 3.6 ± 0.6 µM (Figure 7A).

The ssDNA-binding abilities of the DdrB mutants were assessed by the same fluorescence polarization experiment at a pentamer concentration of 3 µM, and the anisotropy measurements were standardized to the wild-type protein. Under these conditions also using a 20b poly dT ssDNA substrate, all nine mutants were found to be deficient in their ability to bind ssDNA ranging from ∼20–50% of the binding capability of the wild-type protein (Figure 7B). This level of reduced binding is in agreement with what one would expect considering the large number of residues (15 amino acids total) involved in the interface. Mutant E51A was also analysed in this assay and, as expected from EMSA analysis (Figure 5), found to retain full DNA binding activity. Residues R85 and K102 were void of any interactions with ssDNA in the crystal structure, yet substitution of these residues rendered the protein equally deficient in binding as mutation of key residues identified by analysis of the DNA-bound structure. These residues are absolutely conserved amongst all DdrB homologues despite any obvious involvement in structural integrity (Supplementary Figure S2). Together, these findings strongly support the idea that R85 and K102 contribute to an extended ssDNA binding surface delineated by the solvent exposed β-sheet running along the top surface of the DdrB pentamer, which is distinct from the occupied channel surface within the crystal structure (Figure 7C and D).

### Potential mechanisms for DdrB in DNA repair

To date, it has been suggested that DdrB may play a role as both an alternative to SSB in protection of exposed ssDNA (10), and also in promoting annealing of complementary ssDNA strands during the process of SSA (9,12). The crystal structure of DdrB bound to ssDNA illustrates a mode of binding that involves dimerization of two pentamer units. This higher-order structure was further demonstrated to extend to penta-decamers and didecamers in solution (Figure 4). Furthermore, DNA binding studies of wild-type DdrB and a mutant that lacks the ability to form higher-order complexes (E51A) demonstrated that oligomerization facilitates assembly of extended nucleoprotein complexes (Figure 5). Taken together these findings suggest DdrB assembles extended structures able to completely coat ssDNA. By involving direct protein–protein interaction of pentameric units, the cell assures that ssDNA is fully protected, occluding interaction with other proteins and preventing self-association of large stretches of ssDNA. This idea is further supported by the large abundance of DdrB during recovery (8), and also EM studies, which demonstrated that DdrB is able to fully coat circular ssDNA in a manner similar to SSB (10).

**Figure 8.**
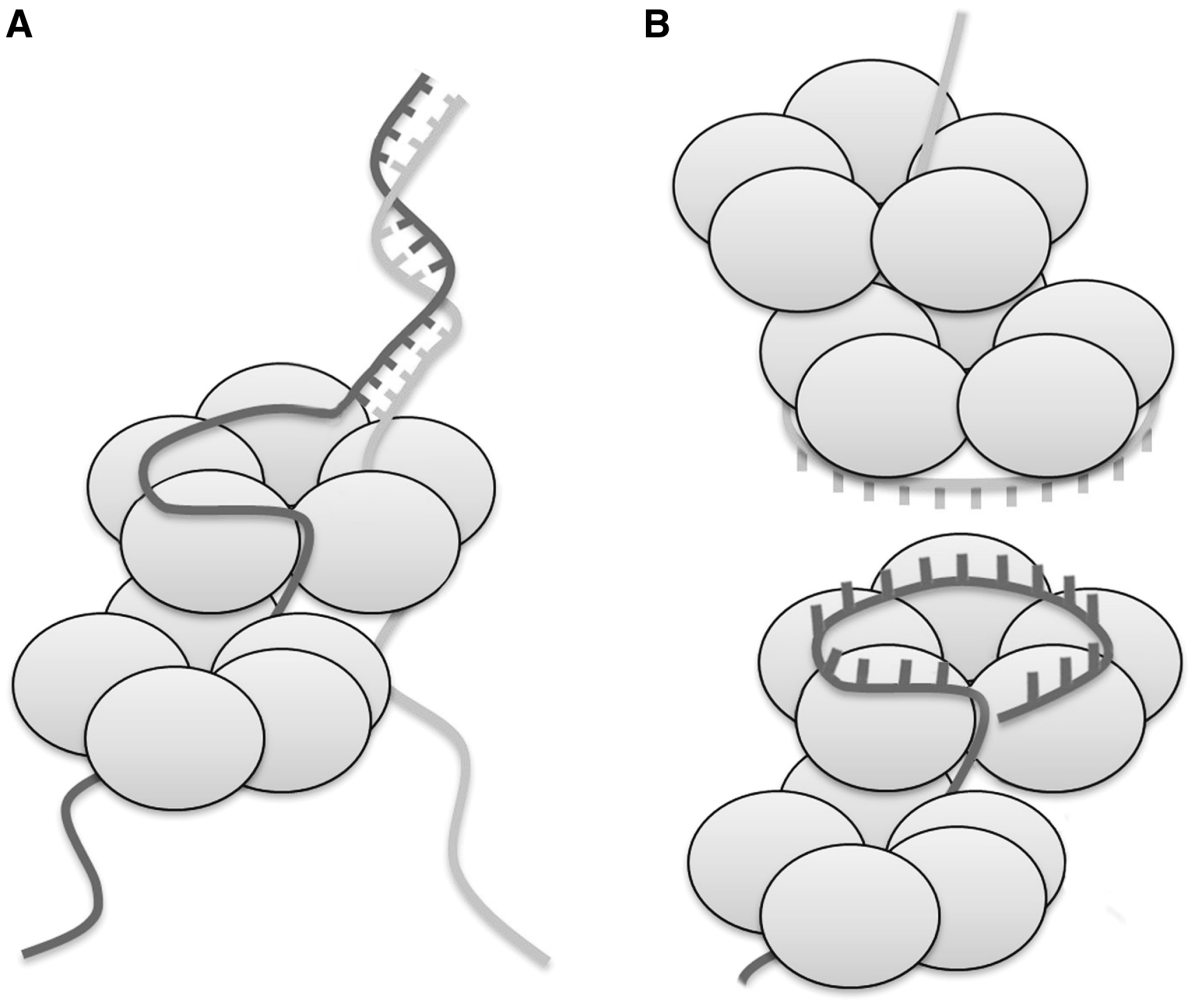
Two models of possible mechanisms for DdrB-mediated ssDNA annealing. (**A**) Two strands of ssDNA thread through different pores on a single DdrB pentamer. One strand is shown interacting with an extended face spanning multiple monomers within the pentamer. Annealing of strands is proposed to be facilitated by the forced juxtaposition of strands at a distinct location on the pentamer. (**B**) A strand of ssDNA is bound to the ‘top’ face of the DdrB pentamer with bases projecting outwards. A second strand, similarly bound to DdrB, senses for complementarity with the outwardly pointed bases.

The limited amount of ssDNA (eight bases) covered by a single DdrB decamer complex, although sufficient to function in coating DNA, is difficult to reconcile with its observed ability to stimulate ssDNA annealing. In addition, binding studies have suggested a much longer DNA length as necessary to fully saturate DdrB (9). An extended DNA binding interface, where ssDNA continues along the top surface of the pentamer involving longer segments of ssDNA, would explain observations from prior binding studies and provide a mechanism for coordinating DdrB’s annealing activity. In this arrangement, DdrB could facilitate annealing by optimally positioning individual strands of ssDNA fed through different pores of a single DdrB pentamer (Figure 8A). Given that a pentamer of DdrB has five of these channels through which it can interact with ssDNA, the actual biological mechanism may be more complicated than the simple model that has been presented. In this scenario, it is not obvious what the role of the extended ‘top’ DNA binding surface would serve and, furthermore, how DdrB would stimulate accurate annealing of DNA. A similar, but more likely, possibility is that DdrB may be acting in a manner similar to what has been proposed for Rad52 (28), in that ssDNA may bind to the top face of the pentamer in such a way that the nucleobases are presented outward, allowing a second strand (also associated with DdrB) to sample the exposed bases for complementarity (Figure 8B). In either scenario, the annealing reaction would be driven by the thermodynamically favourable formation of duplex DNA. During the preparation of this manuscript a report was published describing the EM reconstruction of HSV-1 annealing protein ICP8 in complex with ssDNA (31). The structure suggested a mechanism for ssDNA annealing involving formation of two stacked nonameric ring assemblies with DNA positioned at the interface, reminiscent to what we have proposed for DdrB in Figure 8B.

### Functional similarities between Rad52, DdrB and DdrA

Rad52 is well known for its ability to stimulate ssDNA annealing. DdrA is another protein unique to *Deinococcus* that, unlike DdrB, displays distant sequence similarity to Rad52, and has been suggested to serve a similar function in *D. radiodurans* (32); however, its role in ssDNA annealing has never been reported. Previous experiments have demonstrated that *ddrB* and *ddrA* form separate *recA*-independent epistatis groups, as the double-deletion mutant is more radio-sensitive than either of the single-deletion mutants alone (4). If DdrB serves a role in *D. radiodurans* that is similar to Rad52 in eukaryotes, and DdrA is related by sequence to Rad52, it begs the question of whether DdrA and DdrB are functionally equivalent. If DdrB and DdrA are in fact functional homologues, it stands to reason that deleting one or the other may not have a significant effect on the ability to recover from extensive strand-breakages except in the most extreme cases, and that deletion of both would result in an even more severe phenotype, as was observed (4). Given that both DdrA and DdrB display either functional or sequence similarity to Rad52, the possibility exists that in addition to playing similar roles in alternative pathways, they may be able to complement one another. While the electron microscopy structure of DdrA illustrates a ring-forming assembly similar to both DdrB and Rad52 (32); additional structural information, in the form of a high-resolution crystal structure, would be helpful in evaluating this possibility.

## ACCESSION NUMBERS

PDBID 4HQB (Protein Databank).

## SUPPLEMENTARY DATA

Supplementary Data are available at NAR Online.

## FUNDING

Natural Sciences and Engineering Research Council of Canada through grant [2008R00075 to M.S.J.] and studentship (to S.N.S.M. and Y.M.W.); Offices of Biological and Environmental Research and Basic Energy Sciences of the US Department of Energy; National Center for Research Resources [P41RR012408] and National Institute of General Medical Sciences [P41GM103473] of the National Institutes of Health; Intramural Research Program of the National Institutes Health, the National Institute of Diabetes and Digestive and Kidney Diseases, NIDDK (in part) (to R.G.). Funding for open access charge: National Science and Engineering Research Council of Canada.

*Conflict of interest statement*. None declared.

## Supplementary Material

Supplementary Data
