# Supplementary material for: Crystal structure of the DdrB/ssDNA complex from Deinococcus radiodurans reveals a DNA binding surface involving higher-order oligomeric states
Source: Nucleic Acids Res. 2013 Aug 23;41(21):9934–44. doi: 10.1093/nar/gkt759 (PMC3834827; doi:10.1093/nar/gkt759)
Supplement: Supplementary Data [file supp_41_21_9934__index.html]

Crystal structure of the DdrB/ssDNA complex from Deinococcus radiodurans reveals a DNA binding surface involving higher-order oligomeric states — Supplementary Data 

# Crystal structure of the DdrB/ssDNA complex from *Deinococcus radiodurans* reveals a DNA binding surface involving higher-order oligomeric states

## Supplementary Data

files

**Files in this Data Supplement:**

- Supplementary Data - pdf file
