# Supplementary material for: Crystal structure of the DdrB/ssDNA complex from Deinococcus radiodurans reveals a DNA binding surface involving higher-order oligomeric states
Source: Nucleic Acids Res. 2013 Aug 23;41(21):9934–44. doi: 10.1093/nar/gkt759 (PMC3834827; doi:10.1093/nar/gkt759)
Supplement: Supplementary Data [file supp_gkt759_nar-01695-m-2013-File010.pdf]

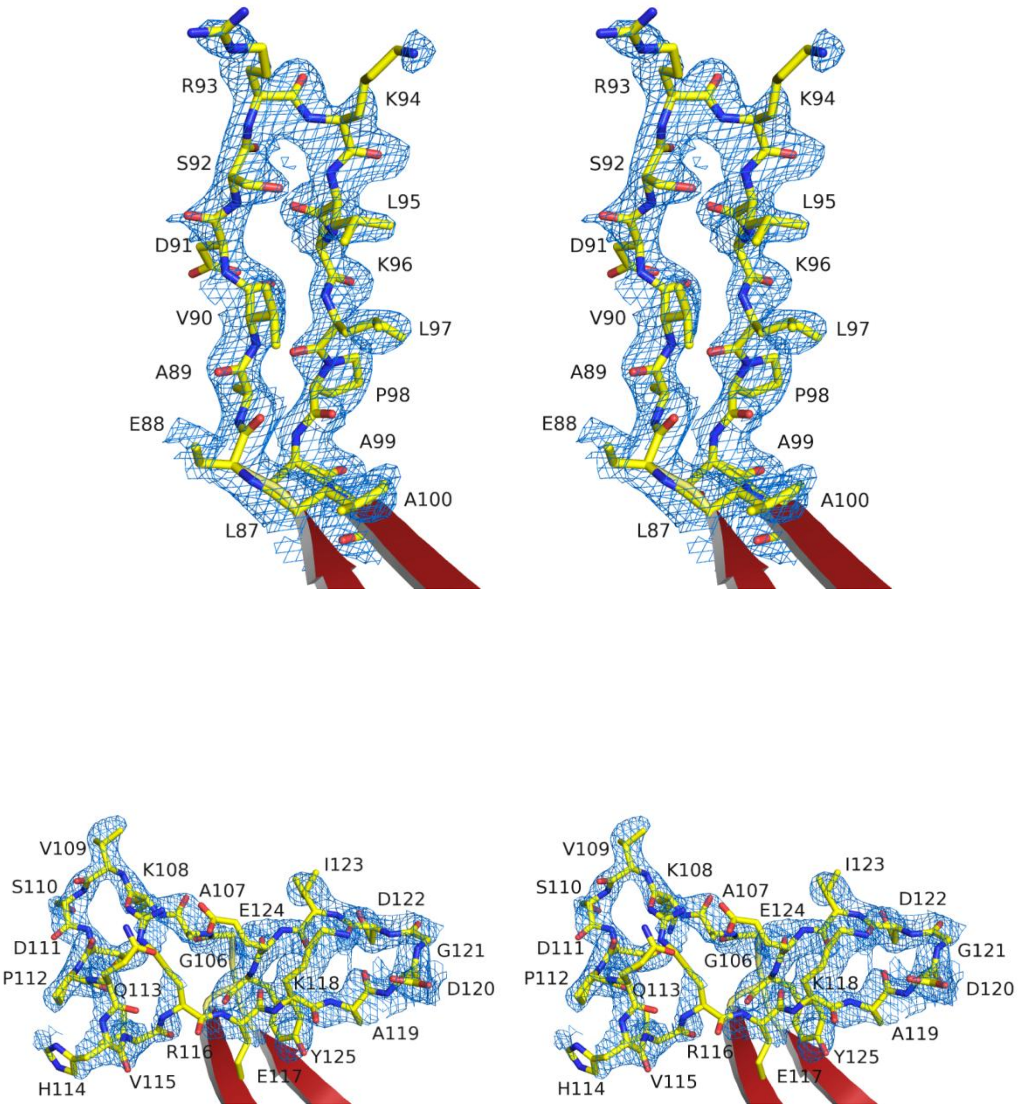

Supplemental Figure 1 – Stereo-images of the electron density of the β6’-β7’ hairpin and $L_{\beta7\text{-}\beta8}$. Simulated annealing OMIT map (2mFo-dFc) of residues making up the hairpin joining strands β6 and β7 (top), and the loop joining β7 and β8 (bottom). Density is contoured to 1.0 σ in PyMol.

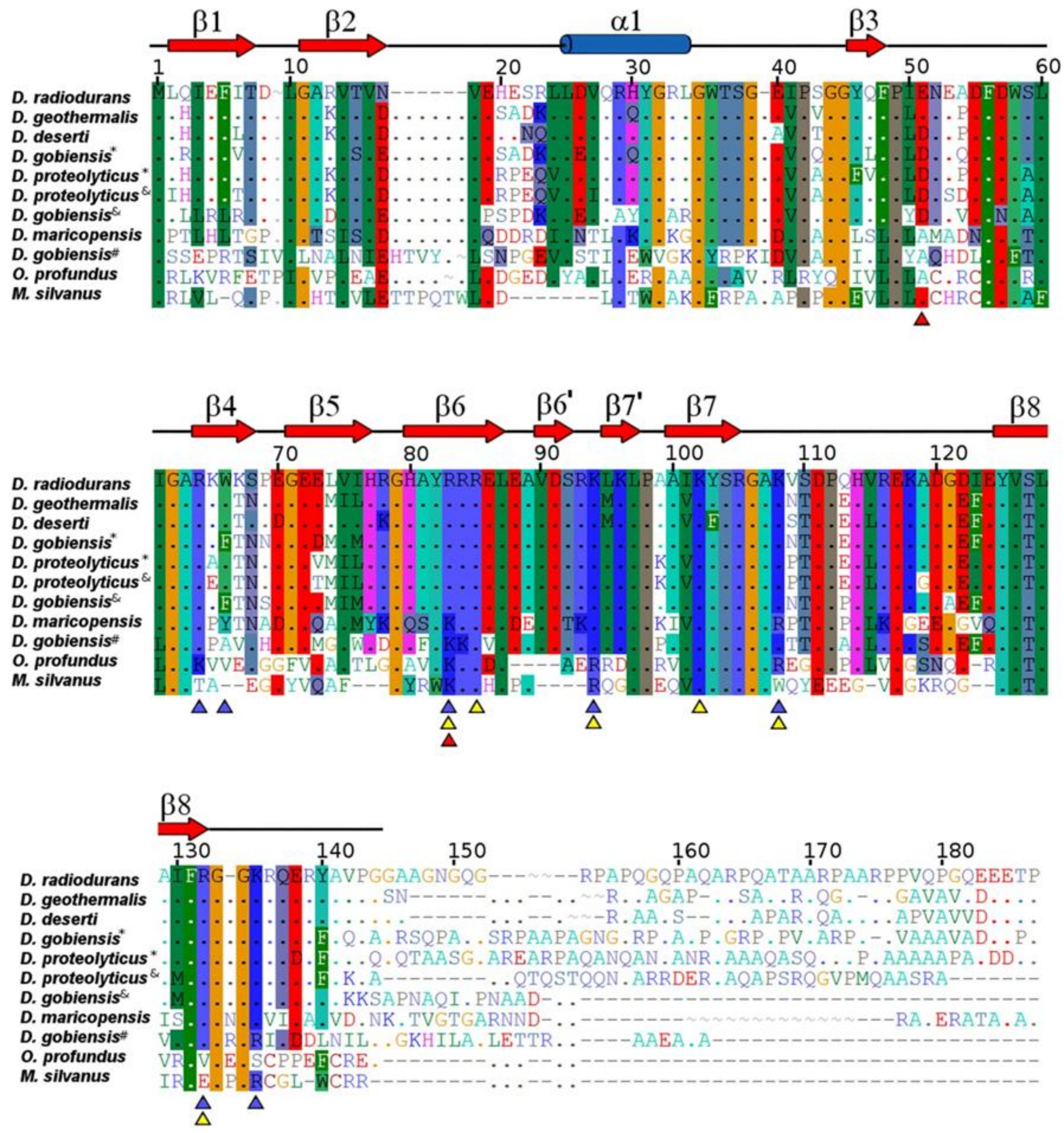

Supplemental Figure 2 – DdrB multiple sequence alignment. 11 homologues of DdrB were aligned using the BLAST server (http://blast.ncbi.nlm.nih.gov/). Species with more than one copy of DdrB are denoted with *(DGo_CA0350, Deipr_1603), &(Deipr_2350, DGo_PC0273) or #(DGo_PB0067). Highly conserved residues are highlighted and coloured by amino acid. Triangles indicate residues involved in: (i) decamer association, E51, R83 (red); (ii) ssDNA interaction, R64, W66, R83, K94, K108, R132, K135 (blue); and (iii) a predicted alternate ssDNA binding surface, R83, R85, K102, K108, R132 (yellow). It should be noted that some residues are likely to participate in both proposed modes of ssDNA interaction and are therefore labelled with more than one triangle.

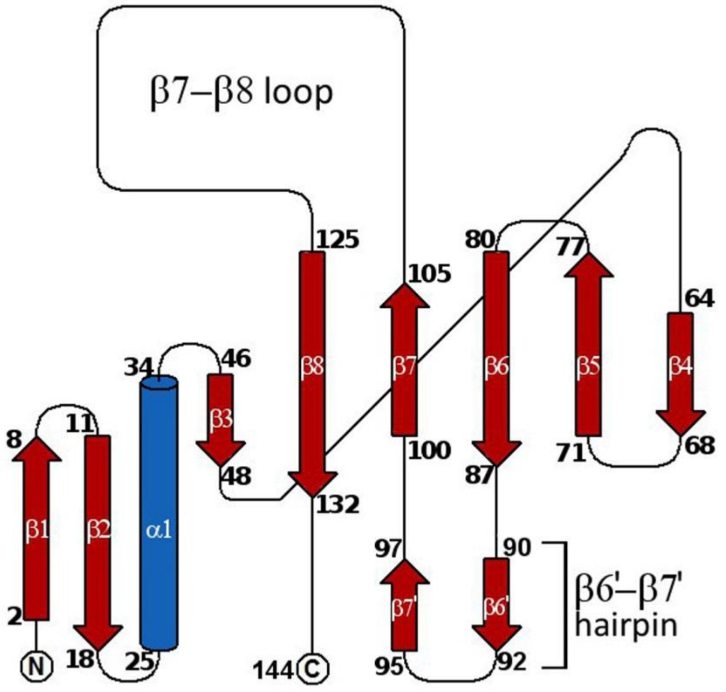

Supplemental Figure 3 – DdrB secondary structure topology. Secondary structure elements are labeled and coloured (red – β-strand, blue – α-helix). Diagram was generated with TopDraw (http://stein.bioch.dundee.ac.uk/~charlie/software/topdraw/).
